# The mosquito holobiont: fresh insight into mosquito-microbiota interactions

**DOI:** 10.1186/s40168-018-0435-2

**Published:** 2018-03-20

**Authors:** Morgane Guégan, Karima Zouache, Colin Démichel, Guillaume Minard, Van Tran Van, Patrick Potier, Patrick Mavingui, Claire Valiente Moro

**Affiliations:** 1 Université de Lyon, Lyon, France; 2 Université Lyon 1, Villeurbanne, France; 3 CNRS, UMR 5557, Ecologie Microbienne, Villeurbanne, France; 4 INRA, UMR1418, Villeurbanne, France; 5 Université de La Réunion, CNRS 9192, INSERM U1187, IRD 249, Unité Mixte Processus Infectieux en Milieu Insulaire Tropical (PIMIT), Plateforme Technologique CYROI, Sainte-Clotilde, La Réunion, France

**Keywords:** Mosquito, Pan-microbiota, Multiple interactions, Symbiosis, Adaptation, Pathogen transmission

## Abstract

The holobiont concept was first developed for coral ecosystems but has been extended to multiple organisms, including plants and other animals. Studies on insect-associated microbial communities have produced strong evidence that symbiotic bacteria play a major role in host biology. However, the understanding of these symbiotic relationships has mainly been limited to phytophagous insects, while the role of host-associated microbiota in haematophagous insect vectors remains largely unexplored. Mosquitoes are a major global public health concern, with a concomitant increase in people at risk of infection. The global emergence and re-emergence of mosquito-borne diseases has led many researchers to study both the mosquito host and its associated microbiota. Although most of these studies have been descriptive, they have led to a broad description of the bacterial communities hosted by mosquito populations. This review describes key advances and progress in the field of the mosquito microbiota research while also encompassing other microbes and the environmental factors driving their composition and diversity. The discussion includes recent findings on the microbiota functional roles and underlines their interactions with the host biology and pathogen transmission. Insight into the ecology of multipartite interactions, we consider that conferring the term holobiont to the mosquito and its microbiota is useful to get a comprehensive understanding of the vector pathosystem functioning so as to be able to develop innovative and efficient novel vector control strategies.

**Electronic supplementary material:**

The online version of this article (10.1186/s40168-018-0435-2) contains supplementary material, which is available to authorized users.

## Background

A holistic view of organisms is necessary to understand the biology of metazoa. The host can no longer be considered as an isolated entity and instead should be considered as a chimera with close interactions with microbial communities. The extent interplay between both partners and its consequences on their evolutionary trajectory has given rise to the holobiont concept (i.e. the host and its community of associated microorganisms as well as their interactions) [1]. Under changing environmental conditions, associated microbiota, i.e. all microorganisms (bacteria, fungi, protists, viruses, etc.), can evolve rapidly and influence the acclimation, adaptation and evolution of host organisms. Changes in microorganism diversity and abundance may have a direct impact on the holobiont’s evolution by modifying the multipartite interaction dynamics.

The holobiont concept—originally developed for coral ecosystems—can also be transposed to other organisms. Most recent advances have shown that human gut microbiota plays a key role in regulating the host’s metabolic functions, immunity, nutrition, physiology and even behaviour [2]. In arthropods, first studies focusing on the molecular mechanisms underlying the role of microbiota such as the host’s immune function, nutrition, physiology and even behaviour concerned phytophagous insects [3]. However, there is still little evidence on the role of microbiota in haematophagous insects of medical importance (pathogen transmission to humans and/or animals) as well as the molecular mechanisms underlying their interactions with the host. The best-known examples concern the mutualistic symbiosis between tsetse flies (Diptera: Glossinidae) and its obligate bacterial symbionts *Wigglesworthia* spp. [4], even though available data on specific *Wolbachia*-mosquitoes associations have considerably increased in the past decades [5]. These bacteria participate in nutrient provisioning, insect fitness, host immunity maturation or pathogen transmission [6]. According to the holobiont concept, an arthropod vector should no longer be considered as an isolated organism but rather as a complex system in which the different partners (host and microbiota) interact.

According to the World Health Organization (WHO), mosquito (Diptera: Culicidae) vectors of arthropod-borne pathogens such as *Anopheles* sp., *Aedes* sp. and *Culex* sp*.* mosquitoes are the greatest threat to public health [7]. *Anopheles* mosquitoes are able to transmit to humans the causal agent of malaria, which is the deadliest vector-borne disease, with about 212 million cases and an estimated 429,000 deaths reported in 2015 [8]. *Culex* sp. mosquitoes are able to transmit both arboviruses and parasites [9] and *Aedes* sp. (mainly *Aedes aegypti* and *Aedes albopictus*) can transmit arboviruses of medical importance to animals and humans, including West Nile (WNV) (Flaviviridae, *Flavivirus*), dengue (DENV) (Flaviviridae, *Flavivirus*), Zika (ZIKV) (Flaviviridae, *Flavivirus*) and chikungunya (CHIKV) (Togaviridae, *Togavirus*) viruses [10, 11]. Dengue is the most serious mosquito-borne viral disease and major chikungunya outbreaks have occurred worldwide over the last decade. Zika virus infection has also recently become a major public health concern, with the global spread of the disease and foetal microcephaly cases arising in women during pregnancy [12]. In addition, some of these mosquito species are invasive and their geographical distribution has been expanding as they take advantage of human activities such as international trade, emphasized by global warming [13, 14]. The lack of effective and preventive treatments against most mosquito-borne diseases restricts control strategies to individual protection and mosquito population control using insecticides. However, intensive and repeated of biocides use leads to the development of mosquito resistance and unwanted effects on non-target species.

There is recent increased interest in studies on mosquito-associated microbiota, which encompass bacteria, fungi, protists and viruses, i.e. both mosquito-specific viruses (MSVs) and the transmitted pathogens. Research on the role of microbial communities in the host biology and pathogen interference has led to the development of new vector control approaches based on the use of “symbiotically” modified mosquitoes [15]. Knowledge on the roles of microbes in the development, physiology or immunity of their hosts, as well as interference with transmitted pathogens, is henceforth essential to be able to develop these alternative strategies. This review summarizes current knowledge on mosquito-associated microbiota. We discuss recent advances and current prospects on the ecology and functions of the mosquito microbiome as well as interactions with the host and transmitted pathogens.

## Influence of the breeding site ecology on mosquito bacterial microbiota

The origin of microbes colonizing mosquitoes and the role of the environment in microbial acquisition are issues that have long been debated [16]. This aspect is essential to define the dynamics of microbial communities in the mosquito holobiont. Most studies carried out to date on microbial acquisition in mosquitoes have been focused mainly on the bacterial component.

**Fig. 1:**
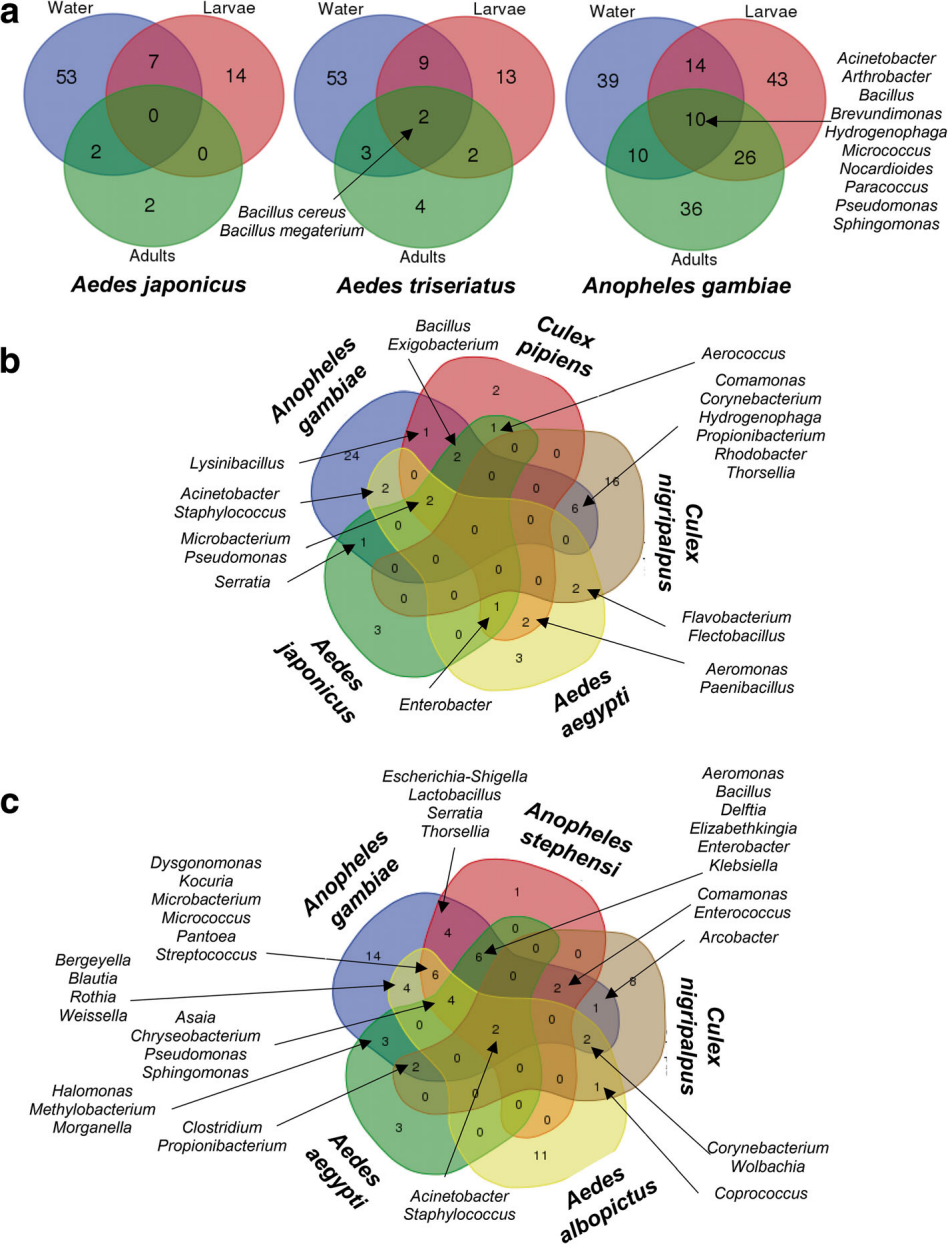
Venn diagrams illustrating overlapping of bacterial composition between mosquito species, development stages and habitats. **a** Number of bacterial taxa specific and common between mosquito larvae, habitats and adults of *Aedes japonicus*, *Aedes triseriatus* and *Anopheles gambiae* [20, 21]. **b** Number of bacterial taxa specific and common to larvae of *Anopheles gambiae*, *Culex pipiens*, *Culex nigripalpus*, *Aedes aegypti* and *Aedes japonicus* [17, 18, 20, 22, 23, 27]. **c** Number of bacterial taxa specific and common to adults of *Anopheles gambiae*, *Anopheles stephensi*, *Culex nigripalpus*, *Aedes albopictus* and *Aedes aegypti* [17, 18, 20, 21, 23, 24, 27, 29–33, 35–37, 39]. An additional table shows in more detail the identification of bacterial species/genera in mosquito species [see Additional file 1]

Recent studies confirmed that a substantial fraction of bacteria colonizing mosquitoes is acquired during the aquatic life stage, through the aquatic larval habitats. The microbial composition and environmental characteristics of breeding sites could partially explain the different colonization patterns of bacteria in immature and adult mosquito stages (Fig. 1). These effects likely accentuate interspecific variations in the microbiota due to the habitat tropism of the different mosquito species. Duguma et al. [17] showed some association patterns between nutrient contents and microbial composition in larval habitats and bacterial communities associated with *Culex nigripalpus* adults. While mosquitoes originating from high-nutrient habitats were associated with members of the Clostridiales order, those from low-nutrient habitats were instead associated with Burkholderiales order members. A specific community profile depending on environmental factors has also been associated with the bacterial composition in *Anopheles gambiae* [18]. Interestingly, the difference in bacterial diversity of larvae from different mosquito species sharing the same breeding site was shown to be lower than that of larvae of similar species living in different collection sites [19]. In *Anopheles coluzzii* and *An. gambiae*, some bacterial communities are shared among fourth instar larvae, water of the larval habitat and adults [20]. Another interspecific comparison indicated that the bacterial communities present in aquatic larval habitats and in larva guts were similar to each other and differed from the bacterial communities of the adult guts [21]. Similarly, only Firmicutes and Actinobacteria phyla were commonly found in both *Ae. aegypti* larvae and aquatic ecosystems, with higher bacterial diversity found in water than in larvae [22]. This suggests that although the bacterial community is acquired from water, the insect’s gut is a more selective habitat for bacteria. This selectivity could be explained by the physicochemical conditions present in the gut (e.g. alkaline pH, redox potential, oxygen level below 5%, etc.) as well as other factors such as the immune response, peristalsis or presence of lytic enzymes or microbial interactions. Moreover, the findings of other studies indicate that some larvae-borne bacteria persist in adults (Fig. 1). Interestingly, the *Thorsellia* genus was detected in both immature (early and late larval instars and pupae) and adult stages in *Culex tarsalis* [23]. In *Ae. albopictus*, some bacteria belonging to Micrococcaceae, Pseudomonadaceae and Staphylococcaceae families are common to larvae, adult males, as well as sugar-fed and blood-fed females [24].

Overall, these results imply that there is a continuum of bacteria from the aquatic environment to immature stages and adult mosquitoes, as shown by the overlap in bacterial composition between water, larvae and adults [20, 25] (Fig. 1). Contrary to previous assumptions [26], bacterial clearance during mosquito metamorphosis from pupae to adults would not be complete, clearly suggesting that a subset of such environmental-acquired bacterial microbiota will intrinsically be part of the holobiont cell components.

## Mosquito core- and pan-microbiota

### From mosquito core-microbiota…

The concept of a core microbiota in mosquitoes (i.e. shared by mosquito populations belonging to the same species) has been addressed in recent years [20]. Generally defined as being a microbial community associated with hosts from a given group (e.g. individual, population, genus, species, etc.), a more flexible definition considering the microbial species most prevalent in the hosts (≥ 90%) has been proposed [27, 28]. Many studies have described core microbiota shared by different adult mosquito populations and/or species collected in distinct geographic areas [see Additional file 1]. For instance, *Pseudomonas*, *Acinetobacter* and *Aeromonas* spp. bacteria were detected in different *Ae. aegypti* populations from Brazil [29]. Likewise, *An. gambiae* populations collected at different sites in Burkina Faso shared *Thorsellia*, *Wolbachia*, *Massilia* and *Acinetobacter* spp. bacteria, which correspond to the most abundant taxa associated with those populations [18]. In Vietnam, *Acinetobacter* was found to be the sole core microbiota constituent of 11 *Anopheles* species [30, 31]. Indigenous and invasive populations of *Ae. albopictus* from Vietnam and France, respectively, also shared core bacterial microbiota, with *Dysgonomonas* being the most prevalent and abundant genus [32]. The tissue tropism of core microbiota was also examined. *Staphylococcus*, *Corynebacterium*, *Geobacillus*, *Micrococcus*, *Acinetobacter* and *Pseudomonas* spp. bacteria are present in male and female *An. gambiae* and *An. coluzzii* reproductive tissues [27]. Tchioffo et al. [33] detected core microbiota composed of *Pseudomonas*, *Comamonas*, *Acinetobacter*, *Rhizobium*, *Burkholderia* and members of the Enterobacteriaceae family in different *Anopheles* mosquito tissues, i.e. ovaries, salivary glands and midgut. Further studies are needed to investigate whether this tissue tropism is correlated with potential core microbiota functions.

### …to mosquito pan-microbiota

As suggested for other organisms, the ecology modulates host-associated microbiota, thus prompting us to apply the pan-microbiota concept to mosquito, e.g. microbiota shared by different mosquito species regardless of their geographic origin [34]. Indeed, environmental factors influence the microbial composition of breeding sites and food resources (plants, sugar, blood). However, as mentioned above, the fact that a common bacterial fraction is shared by different mosquito species at various developmental stages is in favour of the environmental acquisition hypothesis [see Additional file 1]. This repeated-bacterial colonization leads to a stable association between mosquitoes and their environmentally acquired microbiota. For instance, cultivable core microbiota of *Ae. aegypti* and *Ae. albopictus* females in north-eastern India was found to be composed of the same bacterial species, i.e. *Enterobacter cloacae*, *Klebsiella michiganensis*, *Pseudomonas monteilii*, *Bacillus aryabhattai*, *Lysinibacillus fusiformis* and *Staphylococcus hominis* [35]. In* Culex pipiens* and* Culex restuans*, 44% of operational taxonomic units (OTUs) were common in both species [36]. However, bacterial diversity was reportedly lower in distant mosquito species. Only *Bacillus* and *Escherichia*/*Shigella* spp. were common in *Cx. pipiens*, *Culiseta incidens* and *Ochlerotatus sierrensis* [37]. Similarly, *Pseudomonas* and *Wolbachia* were the two genera shared by *Culiseta melanura* and *Coquillettidia perturbans* [38]. A broader study targeting 12 mosquito species in the USA, including *Aedes*, *Anopheles* and *Culex*, revealed similarities among their bacterial communities [see Additional file 1]. These communities were dominated by *Gluconobacter*, *Propionibacterium* and *Staphylococcus* bacterial taxa [39]. Part of the microbiota in adult mosquitoes would thus be acquired from the aquatic larval habitat, while the other fraction would be dependent on food resources, through natural plant-based sugar sources or through blood meals for females. Bacterial composition and diversity are modified subsequently to a sugar- and/or blood-meal which increase inter-individual differences [20]. Notably, blood meal leads to a progressive shift in oxidative conditions in the gut through the modification of microbial communities’ composition and structure [40, 41]. Diversity and composition of the bacterial populations are influenced by both infection status and time after the blood meal in *Ae. albopictus* [42]. The bacterial composition but not its structure is influenced by the blood meal whereas only few taxa varied significantly due to chikungunya virus infection [42]. The bacterial abundance of La Crosse virus (LACV)-infected *Aedes japonicus* and *Aedes triseriatus* increased while richness and evenness of resident fungi decreased [43]. Otherwise, the bacterial abundance is reduced in *Ae. aegypti* infected by DENV [44]. ZIKV infection also modulates the dynamics of the bacterial families *Rhodobacteraceae* and *Desulfuromonadaceae* in *Ae. aegypti*, suggested as potential markers for ZIKV [45]. The associated effects of blood meal and infection tend to accentuate microbiota–mosquito immune interactions, such as immune response as well as redox and detoxifying enzyme metabolisms mentioned below [41]. Similarly, a study conducted by Short et al. [46] showed that part of the host amino acid metabolic pathway, which involves branched chain amino acid degradation, did affect midgut microbial communities in *Ae. aegypti*. This could partly explain variations in the midgut microbiota of mosquitoes in the field.

To summarize, recent reports have confirmed that the ecology of breeding sites drives environmental bacterial acquisition in mosquitoes. Above all, core and pan-microbiota might represent an assemblage fraction of the mosquito that belongs to the extended genome of the mosquito hologenome and contributes to key features of the holobiont. Further studies are necessary to effectively link these core microbes and their genomes with the functions displayed and to determine which host genetic factors govern host-microbe interactions.

## “Neglected” microbes in the mosquito holobiont

### Mosquito virobiota

A novel group of insect-specific viruses has recently been described [47]. In contrast to arthropod-borne viruses which have a dual host tropism (i.e. can replicate in mosquitoes and vertebrates), these insect-specific viruses (ISVs) are host restricted and do not replicate in vertebrate cells. Although most have been discovered in mosquitoes, ISV sequences have also been detected in other haematophagous insects such as sandflies [47]. The cell fusing agent virus (CFAV), Kamiti River virus (KRV) and *Culex* flavivirus (CxFV) were the first endogenous mosquito-specific viruses (MSVs) identified in *Aedes* and *Culex* sp. mosquitoes [see Additional file 1] [48–50].

In the past decade, next-generation sequencing analysis and increasing interest in both microbiome and arbovirus transmission have led to the description of many MSVs [see Additional file 1]. Despite the host restriction, these RNA viruses are related to mosquito-borne viruses (MBVs) and they essentially belong to the Bunyaviridae, Birnaviridae, Flaviviridae, Mesonoviridae, Negoviridae, Reoviridae, Rhabdoviridae and Togaviridae families [51–53]. Most ISVs described to date belong to the Flaviviridae family [54]. Mosquito-specific flaviviruses are divided into two distinct phylogenetic and serologic clades. The first group is closely related to mosquito-borne flaviviruses, including dengue (DENV), yellow fever (YFV) and West Nile (WNV) viruses, while also comprising a dozen virus species [51, 55]. The second one forms a clade distinct from mosquito-borne flaviviruses and includes the previously mentioned viruses CFAV, KRV, CxFV and *Aedes* flavivirus (AeFV), and others [55]. These mosquito-specific flaviviruses are distributed worldwide and have been isolated or detected in diverse mosquito species [see Additional file 1]. For instance, CFAV, KRV, CxFV or AeFV have been isolated or detected in laboratory reared and field-caught *Aedes*, *Anopheles* or *Culex* populations from Africa, Australia, South America or Japan [55]. Mosquito-specific alphaviruses are less diversified as only two viral species have been described [56, 57]. The first one, i.e. the Eilat virus (EILV), was isolated from collected *Anopheles coustani* in Israel [56] and the second one, i.e. the Taï Forest virus, was recently detected in *Culex decens* from Côte d’Ivoire [57].

Despite the high prevalence of MSVs in nature, their acquisition, transmission cycle and maintenance in mosquitoes are poorly known. Like some bacteria, vertical transmission from mother to progeny has been reported. *Culex* and *Aedes* flaviviruses are vertically transmitted in *Cx. pipiens* and *Ae. albopictus*, respectively [58, 59]. However, horizontal transmission could occur as the same MSVs can be detected in different mosquito species from the same geographical area. Both venereal and ectoparasite transmission have been reported. As for phytophagous insects, MSVs could be acquired from the environment and transmitted through infected plants during nectar feeding [51, 53, 60]. MSVs thus have to infect mosquito salivary glands and/or saliva “injected” during feeding. Mosquito flaviviruses CFAV are absent from saliva and salivary glands of *Ae. aegypti* and *Culex annulirostris*, while AeFV was detected in *Ae. albopictus* saliva [61]. Finally, CxFV was only found in *Culex quinquefasciatus* saliva bi-infected with WNV [61]. The Eilat alphavirus was detected in salivary glands of *Ae. aegypti*, *Ae. albopictus* and *Cx. quinquefasciatus* [61]. As no horizontal transmission was detected between *Cx. pipiens* mosquitoes infected or not by CxFv and reared in the same cage with common food resources [62], further investigations are required to corroborate these horizontal modes of transmission. Temperature could modulate their prevalence and/or maintenance in field populations since mosquitoes are ectotherms and some MSVs are temperature sensitive [63].

Reverse genetic tools have revealed that restrictions occurred during cell entry and replication steps and during viral assembly for Eilat and Niénokoué viruses, respectively [56, 64, 65]. Mammal innate immunity could also explain host restriction, as demonstrated for KRV, which can complete its viral replication cycle in cells deficient in some interferon regulatory factors [66].

### Mosquito-associated eukaryotes

In addition to viruses and bacteria, the mosquito microbiota is also composed of fungal (mycobiota) and protist communities [see Additional file 1]. However, few studies have focused on these communities in mosquitoes. Recently, Belda et al. [67] developed an efficient method that enabled the identification of eukaryotic microbiota associated with *Anopheles* mosquito larvae. Ichthyosporeans of the Pseudoperkinsus group were the most abundant protist members. Steyn et al. [68] also provided the first description of yeast microbiota of *Cx. pipiens* and *Culex theileri* larvae. The authors identified *Candida*, *Cryptococcus*, *Galactomyces*, *Hannaella*, *Meyerozyma*, *Pichia*, *Rhodosporidium*, *Rhodotorula*, *Trichosporon* and *Wickerhamomyces* genera. Apart from non-pathogenic fungi, mosquitoes also harbor yeasts of clinical importance. A recent study reported the isolation of the opportunistic pathogen *Candida parapsilosis* from different developmental stages and organs of several laboratory-reared mosquito species, including *An. gambiae*, *An. stephensi*, *Cx. quinquefasciatus*, *Ae. albopictus* and *Ae. aegypti* [69]. Moreover, culturable yeast microbiota analysis from wild *Cx. pipiens* and *Cx. theileri* mosquito larvae revealed the presence of clinically relevant species, including the well-known opportunistic human pathogen *Candida albicans*.

Overall, there has been much consideration of bacterial microbiota in the holobiont concept as it represents the abundant fraction of the host microbiota and consequently has been shown to impact the extended phenotype. The advent of high-throughput sequencing methods has made it possible to describe other communities of microbes including viruses and protists. Some of them, even at lower abundances, established notable interactions with their host. In mammals, retroviral genes are thought to be involved in the placenta formation [70]. We provided here some published data on virobiota and eukaryotic microbiota associated with mosquitoes. We encourage experimental evolutionary and functional researches to be performed on this neglected microbiota and we anticipate, in light of what was demonstrated with bacterial microbiota, that it will build a foundation to extend the list of microbial taxa involved in the mosquito holobiont.

## Microbiome-mosquito interactions and mosquito holobiont success

As previously mentioned, most studies on mosquito microbiota have been descriptive and focused on bacteria with the aim of drawing up an inventory of the microbial communities [see Additional file 1] and their variation factors. While recent studies have demonstrated interactions between bacteria and mosquitoes as well as between fungi and mosquitoes, the role of MSVs in the host biology remains to be determined. Cytophatic effects have been observed in cell cultures, but it is still unknown whether and how MSVs influence host life history traits (lifespan, fecundity, oviposition).

### Growth and larval development

**Fig. 2:**
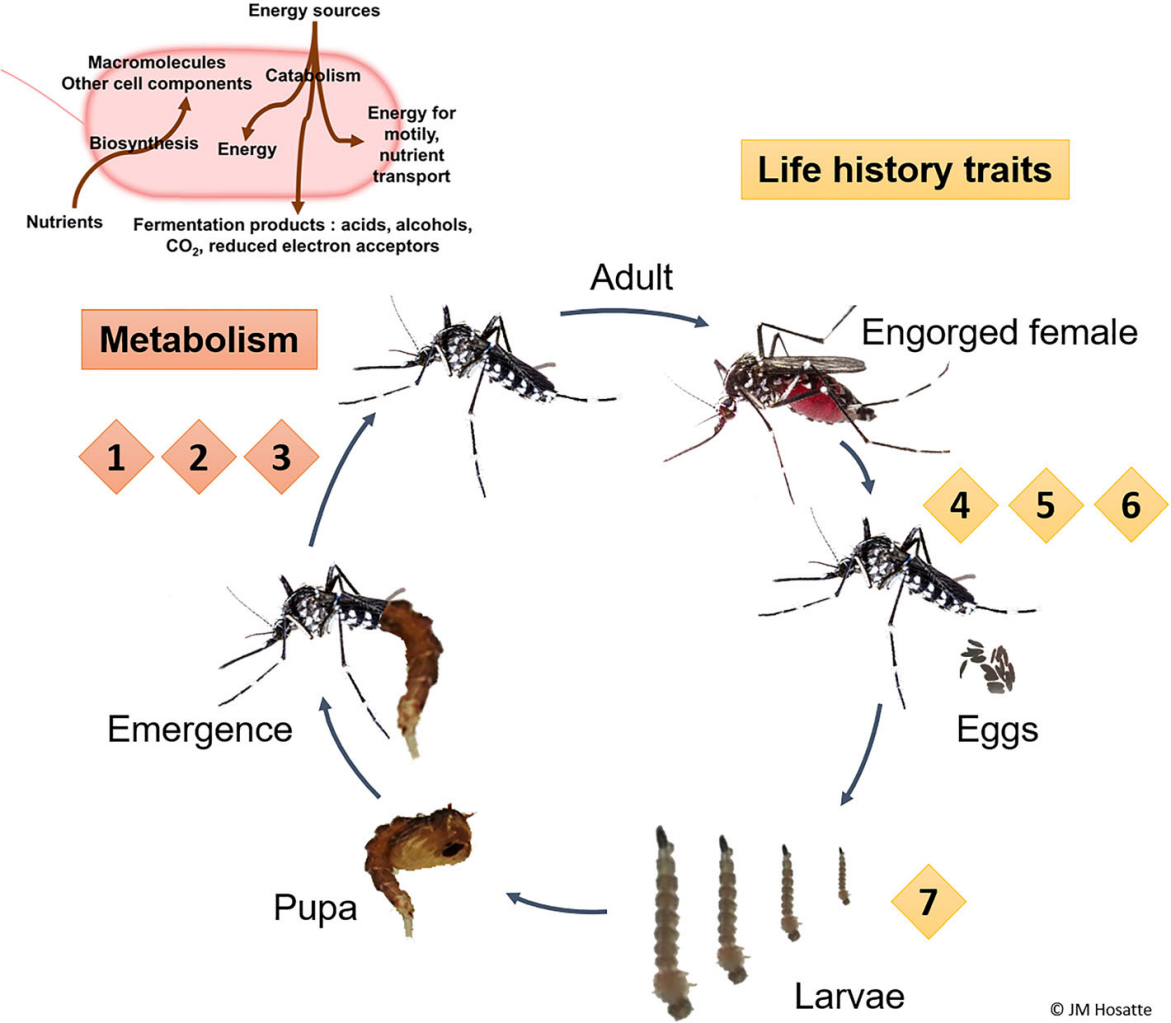
Putative functions of mosquito-associated microbiota (bacteria and fungi). Functions related to metabolism are indicated in orange and those related to life history traits are indicated in yellow: (1) Blood digestion (*Acinetobacter*, *Pantoea*, *Enterobacter*, *Dysgonomonas*), (2) Sugar digestion (*Acinetobacter*, *Elizabethkingia*, *Thorsellia*, Sphingomonadaceae family, *Meyerozyma*), (3) Supply of vitamins and amino acids (*Dysgonomonas*, *Klebsiella*, *Aeromonas*, *Saccharomyces cerevisiae*), (4) Survival (*Escherichia coli*, *Beauveria bassiana*), (5) Mediating oviposition site choice (*Klebsiella*, *Aeromonas*), (6) Egg production (*Comamonas*), (7) Larval development (*Acinetobacter*, *Asaia*, *Aeromonas*, *Chryseobacterium*, *Paenibacillus*, *Aquitalea*, *Escherichia coli*) [19, 25, 68, 71–75, 77, 80]. Pictures from JM Hosatte, with permission

In recent years, major studies have focused on the role of microbes in mosquito development (Fig. 2). One of the most relevant examples is about the *Asaia* genus, which is closely associated with *An. stephensi* and is one of the principal members of its microbiota. Up- and downregulation of genes involved in cuticle synthesis in *Asaia*-infected larvae promotes the insect’s growth (size and development stages) [71]. Similarly, both bacteria (*Klebsiella* and *Aeromonas)* and yeasts *(Saccharomyces cerevisiae)* promote *Cx. pipiens* development [72]. Another study showed that the survival and pupation of *Cx. pipiens* larvae were negatively impacted when they were fed with yeast isolates compared to fish food [68].

Besides experimental infections, antibiotic treatments are the main approaches used to manipulate mosquito bacterial communities for the purpose of studying the functional roles of insect microbiota. However, these methods fail to eliminate all the bacteria. An alternative approach was therefore developed to generate axenic (i.e. without microorganisms) mosquitoes using a combination of ethanol and bleach to sterilize the egg surface. Gnotobiotic (i.e. with known microbiota) individuals can thus be obtained via inoculation of a given bacterium [25] or other microbes. Recent studies using this experimental approach, together with functional assays, clearly demonstrated that larvae rely on bacteria for their development. All axenic *Ae. aegypti*, *An. gambiae* and *Aedes atropalpus* larvae fed with a standard sterile diet died during the first larval instar [25]. However, mono-associated inoculation of *Acinetobacter*, *Aeromonas*, *Aquitalea*, *Chryseobacterium* or *Paenibacillus* restored *Ae. aegypti* larval development. Likewise, *Ae. albopictus* and *Cx. quinquefasciatus* require gut colonization by living bacteria for their development [19]. Overall, these results suggest that mosquitoes do not rely on specific bacterial taxa for their development but rather on exchangeable symbiont combinations that could be acquired through larval aquatic habitats.

Some mechanisms involved in larval development have been described. Genes involved in nutrient acquisition, metabolism and stress responses are differentially expressed in the first axenic *Ae. aegypti* larval stage compared to conventional and gnotobiotic ones, suggesting a putative role of gut bacteria in nutrient acquisition and/or assimilation after hatching [73]. Mechanisms were detected with *Ae. aegypti* gnotobiotic larvae colonized by different *E. coli* mutants [74]. The cytochrome bd oxidase gene appeared to be a key component in this interaction by reducing the gut oxygen level. Consequently, hypoxia induces the stabilization of hypoxia-induced transcription factors (HIFs) that enables larval growth and ecdysone-induced molting [74, 75]. To summarize, larvae acquire some of their bacterial microbiota from aquatic habitats and these bacteria contribute to the host development, survival and pupation. Any disturbance of the microbial community in the larval aquatic habitat could therefore impact the mosquito biology and ecology [76].

### Egg production and oviposition

Coon et al. [77] demonstrated the contribution of gut bacterial microbiota in egg production by *Ae. aegypti* and *Ae. atropalpus* while comparing gnotobiotic larvae to their axenic relatives (Fig. 2). *Ae. atropalpus* can produce their first clutch of eggs without blood-feeding and depends on specific members of their gut microbiota to produce eggs whereas a blood meal is mandatory for *Ae. aegypti*. *Ae. atropalpus* microbiota probably provides nutrient reserves during larval development which are necessary for the first clutch in the absence of a blood meal. Interestingly, *Cx. pipiens* females were shown to select media containing *Klebsiella* and *Aeromonas* bacteria for oviposition [72].

### Mosquito pathogens

Nowadays, *Bacillus thuringiensis subsp. israelensis* (Bti) producing Cry, Cyt, Vip and Sip insecticidal proteins is a safer alternative to chemical insecticides. However, its repeated and intensive use was found to generate strong selection pressure that could promote Bti resistance. A reduction in bacterial microbiota diversity in *An. stephensi* larvae has been shown to increase their susceptibility to Bti [78]. This finding differs from what was previously known in other insect models where microbiota was a key component in Bti efficiency [79]. In addition, the microaerophilic conditions of the larval gut would also favour the use of insecticidal proteins as a source of nitrogen by bacteria [78]. Intestinal bacteria would increase larval resistance through Bti toxin degradation. There has been recent increased interest in the identification of mosquito-killing fungi to tackle insecticide resistance. It was recently shown that the pathogenic fungus *Beauveria bassiana* could interact with gut bacterial microbiota and accelerate *Anopheles* mosquito death [80]. The fungus induced an increase of the opportunistic pathogenic bacterium *Serratia marcescens* density that lead to its dissemination in the haemocoel and promote death of their host [80].

Published data reported here illustrate that hypothesis- and experimentally- driven researches are key elements to demonstrate the contribution of bacterial microbiota in extended phenotypes of the mosquito holobiont. Notably, the manipulation of microbial assemblages allows to deduce their important impact on the mosquito life history traits and somehow which host-genes were modulated.

## Mosquito-microbiota co-evolution

Recent studies have highlighted host-microbiota phylosymbiosis, i.e. a congruency between the host phylogeny and the divergence in its associated microbial community composition [81, 82]. A field study conducted by Novakova et al. [82] in 11 mosquito species from Canada revealed congruency between the phylogeny of hosts and differences in their associated bacterial communities. However, this co-evolutionary pattern was not observed for all related host species. Indeed, in standard laboratory conditions, *Ae. aegypti* showed more divergent microbiota from the closely related species *Ae. atropalpus* than from the distantly related species *An. gambiae* [25]. Conversely, a congruence pattern in microbial communities was also observed in species belonging to the same subgroup. Analysis of gut bacterial microbiota revealed a strong similarity between *Ae. albopictus* and a cryptic species living in sympatry in Vietnam [81]. Those findings could possibly be explained by recent host-microbe co-adaptation. Additional studies are necessary to clarify the influence of local environmental parameters and host genotypes on the mosquito microbiota composition. Evolutionary prospects of *Wolbachia*-mosquito associations have also recently been highlighted. Besides, horizontal gene transfers (HGTs) were described between *Aedes* mosquitoes and *Wolbachia* [83, 84]. The evolution of *Wolbachia*-mosquito associations raises the question of the outcome of bacterial-to-mosquito HGTs on adaptive capacities of the holobiont or/and mosquito vector competence, as *Wolbachia* can modulate the transmission of some pathogens depending on the mosquito species [5]. The adaptive capacities of the holobiont could also be impacted by functional bacterial HGTs. This event was demonstrated for the coffee-crop insect that acquired a gene encoding a polysaccharide enzyme from bacteria belonging to Bacilli class through HGT, that could extend its host-plant range and so promote its adaptation to new ecological niches [85].

Likewise, phylogenetic studies have highlighted that some MSVs belonging to Bunyaviridae and Flaviviridae families have co-evolved and diversified with their mosquito host [63, 86]. Transovarial transmission as well as the detection of integrated viral sequences in mosquito genomes (i.e. endogenous viral elements [EVEs]) [87] supports the hypothesis of an “ancient” association and possible co-evolution. A description of new lineages of mosquito-specific bunyaviruses associated with phylogenetic ancestral reconstruction indicated that pathogenic bunyaviruses have evolved from an arthropod-specific ancestor [63]. Those data suggest possible adaptation of MSVs to vertebrates, following a spillover phenomenon (i.e. crossing of species barrier), and the emergence of other pathogenic viruses.

In this evolutionary context, in addition to bacterial microbiota, attention should be paid on MSVs as they may represent drivers of biological traits linked to the ability of the mosquito holobiont to transmit or not pathogens (see below).

## Microbial interactions

### Intra-microbial community interactions

Microbial interactions shape mosquito bacterial diversity and structure. To date, few data on interactions of gut microbes are available. Most of studies on microbe-microbe interactions focused on the influence of *Wolbachia* on the microbiome, or vice versa [88]. *Wolbachia* are intracellular, vertically transmitted bacterial symbionts that naturally infect many mosquito species and are known to manipulate their reproduction through cytoplasmic incompatibility (e.g. the offspring of infected males and uninfected females are not viable) [89]. In *Anopheles* mosquitoes, the identification of new factors modulating *Wolbachia* transmission in artificially transfected adults highlighted the importance of native mosquito microbiota and interaction with *Wolbachia* [90]. The disturbance of bacterial microbiota by antibiotic treatment reduced the ability of *Anopheles* to transmit the *Wolbachia w*AlbB strain (from *Ae. albopictus*) to offspring. Additional experiments combining high-throughput sequencing and oral infection of bacteria revealed that their native microbiota, especially *Asaia*, impeded vertical transmission of *Wolbachia*. This co-exclusion pattern between *Wolbachia* and *Asaia* is also found in *Ae. albopictus* and *Cx. quinquefasciatus* naturally bi-infected by both bacteria for which *Asaia* tissue tropism is restrained to the gut. Conversely, *Asaia* is also able to colonize reproductive organs and salivary glands in species uninfected by *Wolbachia* such as *An. gambiae*, *An. stephensi* and *Ae. aegypti* [91]. These observations suggest co-exclusion or competition between the two bacterial genera for reproductive organ colonization.

Bacteria could also interact with mosquito-specific viruses but so far only *Wolbachia*-ISV interactions have been considered [92]. An *Ae. aegypti* derived-cell line (Aag2) transfected with a *Drosophila melanogaster*-derived *Wolbachia* strain (*w*Melpop) was infected with the mosquito-specific CFAV flavivirus or with the Phasi Charoen-like bunyavirus. Molecular analysis revealed inhibition only for CFAV in Aag2-*w*Melpop cells, possibly related to the production of CFAV-specific small RNAs [92]. Indeed, MSVs could induce the RNA interference (RNAi) pathway by producing small interfering RNAs (siRNAs), as demonstrated for MBV [93]. The extent to which these interactions are genotype-by-genotype dependant is unknown. Moreover, it is also unknown whether *Wolbachia* can inhibit other single negative- or positive-strand RNA viruses using in vitro and/or in vivo systems. *Wolbachia* and MSV interactions in mosquitoes are nevertheless conceivable, as both are vertically transmitted intracellular organisms, suggesting co-localisation in reproductive organs. Their co-localisation at the cellular level still needs to be determined as it could impact the maintenance and transmission of both microbes in field bi-infected populations.

### Microbial interference with transmitted pathogens

Mosquito infections with pathogens, including transmitted pathogens, trigger a complex crosstalk between different metabolic and immune pathways. Innate immune systems such as immune deficiency (Imd), the Toll, Janus kinases and signal transducers and activators of transcription (JAK-STAT) are activated in response to diverse microbes (viruses, bacteria, fungi or parasites) whilst RNAi modulates virus replication [41, 94, 95]. This general immune homeostatic response can be associated with the induction of autophagy, apoptosis as well as oxidative stress [40]. Transmitted pathogens can alter metabolisms through the modulation of stress-inducible genes involved for instance in redox and detoxifying enzyme metabolisms. However, molecular responses to transmitted pathogens are more complex if we consider the holobiont. The interplay between microbiota and mosquito immune system may result in an enhanced synergistic effect on the expression of effector molecules of the mosquito immune system, as previously reviewed [96].

#### Bacteria-mosquito-borne pathogen interference

As shown in Table 1, recent reports have stressed the importance of microbiota in the modulation of vector competence (ability of a susceptible mosquito to get infected by a pathogen, to support the pathogen’s replication and/or development and to transmit the pathogen to a susceptible vertebrate host)—this phenomenon is called microbial interference [97]. Multipartite interactions between the pathogen, the mosquito and its bacterial microbiota have become a major target for developing new control strategies in order to stop pathogen transmission and related epidemics.

**Table 1:** Examples of microbial interference between microbiota and vector-borne pathogens

Pathogen		Mosquito	Microorganism	Interference	References
Arboviruses	Dengue virus	*Aedes aegypti*	*Serratia odorifera*	Enhances susceptibility to the virus	[115]
			*Chromobacterium*	Increases infection resistance Antiviral activity Immune elicitor	[99]
			Enterobacteriaceae, Esp_ivi isolate, alternatively *Salmonella*, *Escherichia* or *Shigella*	Decreases antibacterial activity Reduces virus dissemination titer	[113]
			*Wolbachia*	Reduces susceptibility to the virus	[153]
	West Nile virus	*Culex pipiens* Colorado strain	*Culex* Flavivirus (CxFV)	Reduces virus dissemination	[62]
		*Culex quinquefasciatus* Honduras strain	*Culex* Flavivirus (CxFV)	Enhances virus dissemination	[132]
		*Culex quinquefasciatus*	Nhumirim virus (NHUV)	Reduces virus infection	[135]
	Chikungunya virus	*Aedes aegypti*	*Serratia odorifera*	Enhances susceptibility to the virus	[115]
			*Eilat* virus	Reduces virus replication and dissemination	[64]
	La Crosse virus	*Aedes albopictus*	*Enterobacter ludwigii*	Antiviral activity	[116]
			*Pseudomonas rhodesiae*	Antiviral activity	[116]
			*Vagococcus salmoninarium*	Antiviral activity	[116]
Parasites	*Plasmodium yoelii*	*Anopheles dirus*	Bacterial microbiota	Protects against infection by regulating *tep1* expression	[109]
	*Plasmodium falciparum*	*Anopheles gambiae*	*Chromobacterium*	Increases infection resistance by forming a protective biofilm against parasite	[99]
			*Enterobacter*	Inhibits parasite development by stimulating oxydative stress	[100, 101]
			*Escherichia coli*	Reduces infection prevalence and intensity Reduces parasite development	[98]
			*Pseudomonas stutzeri*	Reduces infection prevalence and intensity	[98]
			*Serratia*	Protects against infection	[103]
			*Serratia marcescens*	Reduces infection prevalence and intensity Actives IMD/REL2 immune pathway	[98, 106]
			*Penicillium chrysogenum*	Enhances susceptibility to the parasite	[130]
	*Plasmodium berghei*	*Anopheles stephensi*	*Asaia bogorensis*	Inhibits parasite development by secreting a siderophore receptor protein and a YVTN beta-propeller repeat protein	[140]
			*Wickerhamomyces anomalus*	Anti-*Plasmodium* activity	[129]

In *An*. *gambiae* mosquitoes, the presence of *Escherichia coli*, *Serratia marcescens* or *Pseudomonas stutzeri* resulted in a significant reduction in the prevalence and intensity of *Plasmodium falciparum* infection [98]. The abundance of *Serratia* was positively correlated with *P. falciparum* infection in both the midgut and salivary glands, suggesting a potential interaction between bacteria and the malaria parasite. The bacterium *Chromobacterium* was shown to increase *An*. *gambiae* resistance against *P. falciparum* [99]. It was suggested that the underlying mechanism behind interference was the production of cyanide by the bacterium. *An. gambiae* mosquitoes are also naturally colonized by the *Enterobacter* Esp_Z bacterial strain which inhibits the development of *Plasmodium* parasites prior to midgut colonization [100]. Specific genes associated with reactive oxygen species (ROS) production were found to be involved in mosquito midgut colonization by Esp_Z bacteria [101]. Recent studies have demonstrated the ability of gut bacteria to produce antiparasitic effectors that inhibit parasite growth. For instance, *Chromobacterium* was suggested to be a powerful immune elicitor since it increases mosquito immune gene expression. This feature, combined with its ability to rapidly invade the mosquito gut and reduce the lifespan of immature stages and adult mosquitoes, makes this bacterium a promising candidate for vector control applications [99]. Another study showed that the *E. coli* clone 444ST95 previously isolated from *Anopheles* mosquito midgut is able to markedly decrease the survival of these mosquitoes as well as the development of their *Plasmodium* parasites [102]. Hemolysin F or other toxins released by the bacterium are virulence factors associated with this effect [102]. Finally, a positive correlation between intensive antibiotic therapy in humans and increased risk of malaria transmission by *An*. *gambiae* mosquitoes has been suggested [103]. Indeed, antibiotics ingested by humans and circulating in their blood would enhance the susceptibility of blood-sucking *An. gambiae* females to malaria infection by disturbing their gut microbiota [103]. Despite the overall decrease in the microbial load in the mosquito, some specific changes have occurred in the microbial community, including a reduction in *Serratia* density, associated with an increase in *Asaia *abundance. Gendrin et al. [104] subsequently showed that critical parameters for the mosquito vector capacity, such as lifespan, permissiveness to *P. falciparum*, the mosquito microbiota composition and gut homeostasis were specific to the antibiotic treatment used. Moreover, microbiota disruption is closely related to gut homeostasis regulation [105]. For instance, the peritrophic matrix (PM) is a membrane that physically separates the blood meal from epithelium cells and plays a key role in regulation of mosquito gut homeostasis. Rodgers et al. [105] demonstrated that PM synthesis and integrity are related to gut microbiota. Importantly, microbial metabolites trigger the host oxidative response in mosquitoes, while maintaining redox homeostasis in the midgut [40].

As previously mentioned, the immune system is enhanced during microbial infections in mosquitoes. Stathopoulos et al. [106] characterized the molecular processes driving the mosquito immune response following infection by the enterobacterium *S. marcescens* as well as its consequences on transmission of the parasite. Peptidoglycan recognition proteins (PGRPs) are key regulators of the innate immune response [107]. These proteins specifically recognize microbe-associated molecular patterns (MAMPs) and therefore are influenced by microbiota variations. *S. marcescens* infections were found to result in the activation of some host genes, including PGRP-LC which activates the IMD/REL2 immune pathway involved in a reduction in *Plasmodium* infection. The authors also showed that some effectors of *Anopheles* immunity could impact the microbiota composition and load [107]. Similarly, Gendrin et al. [108] demonstrated that PGRP are important regulators of mosquito epithelial immunity and vector competence. PGRP-LA and PGRP-S2/ PGRP-S3 would be involved in the antiparasitic defense system, while PGRP-LB would promote mosquito permissiveness to *P. falciparum*. Immune system modulation by gut microbiota has also been demonstrated in *Anopheles dirus* mosquitoes naturally resistant to *Plasmodium yoelii* infection [109]. Its microbiota regulates the expression of a thioester-containing protein 1 (TEP1) following parasite infection. *tep1* inactivation prevents microbiota from protecting the mosquito against parasitic infections [109]. This result suggests an important role of TEP1 related to microbiota in the refractoriness to *P. yoelii* infection. The immune regulation through microRNAs (miRNAs) can also modulate anti-*Plasmodium* defense and midgut microbiota [110]. The use of transgenic mosquitoes engineering to express miRNAs targeting endogenous-miRNAs offers new perspectives for the development of alternative malaria control.

There is abundant literature on *Wolbachia*-mediated interference of arbovirus transmission, as previously reviewed [111]. Here we will mainly focus on mosquito-borne pathogen interference with other members of bacterial microbiota. Indeed, few studies have examined the role of the microbiota in the modulation of arbovirus replication and transmission in mosquitoes. Interference mechanisms evidenced include production of bacterial metabolites with an anti-viral activity or nutrient competition between arbovirus and resident microbiota. As for *Plasmodium*, the bacterium *Chromobacterium* produces a metabolite with an anti-DENV activity in *Ae. aegypti* [99]*.* By comparing untreated and antibiotic-treated mosquitoes, Audsley et al. [112] demonstrated that the microbiota composition was not essential for blocking DENV in laboratory-reared *Ae. aegypti*. However, it seems that this assessment cannot be generalized since larval exposure to an Enterobacterium isolate Esp_ivi (genus-level classification undetermined, alternatively *Salmonella*, *Escherichia* or *Shigella*) resulted in decreased antibacterial activity in the hemolymph of *Ae. aegypti* females and reduced DENV dissemination titers [113]. Conversely, *Serratia odorifera* enhanced the susceptibility of *Aedes* mosquitoes to dengue and chikungunya viruses [114, 115]. A recent study also showed that *Enterobacter ludwigii*, *Pseudomonas rhodesiae* and *Vagococcus salmoninarium* isolated from *Ae. albopictus* could have an anti-viral effect on the La Crosse virus in vitro [116]. Interestingly, Novakova et al. [82] demonstrated that bacterial microbiota could be an important factor in the variability of vector competence in mosquitoes for WNV. The findings of other studies have also confirmed this observation. *Ae. albopictus* populations from France were shown to be more efficient in chikungunya virus dissemination compared to Vietnamese autochthonous relatives [117, 118]. In parallel, these invasive populations exhibited a reduction in their gut bacterial diversity compared to the Vietnamese populations [32].

Evidences underscore the importance of RNAi pathways in antiviral defense by the modulation of the expression of host or virus RNA-derived small RNAs, including siRNAs, PIWI interacting RNAs (piRNAs) and miRNAs. The siRNAs are the predominant virus-derived RNAs detected in infected mosquitoes, but piRNAs can also be detected in late/persistent infection [119, 120]. Zika virus modulates expression of both virus-derived siRNAs and piRNAs and host-derived miRNAs in *Ae. aegypti* [121]. Silencing of RNAi enzyme effectors such as Argonaute-2 (Ago-2) or Dicer 2/ R2D2 complex can promote flaviviruses (DENV) or alphaviruses (CHIKV, O'nyong'nyong virus [ONNV], Sindbis virus [SINV]) replication and/or transmission by *Aedes* and *Anopheles* mosquitoes [119, 120]. In a same way, RNAi-mediated knockdown of Imd and JAK-STAT pathways increased DENV replication in some *Ae. aegypti* lines [119]. Arboviruses can also induce protein synthesis involved in ROS production, carbohydrate or lipid metabolisms. In particular, midgut infection by DENV-2 and CHIKV triggered an antioxidant response through the production of proteins involved in detoxification. Other anti-viral responses such as apoptosis and autophagy can also contribute to innate antiviral immunity [119, 120], but detailed mechanisms involved remains poorly understood. Overall, the relative implications and crosstalk of these metabolic and immune pathways remain to be clarified and seem to be dependent on multiple factors, notably the transmitted virus-mosquito combination of the holobiont.

These crosstalk pathways become more complex as mosquito immune responses to transmitted pathogens can influence resident microbiota and vice versa. Only few data on anti-arbovirus responses in the context of holobiont are available. One of the most documented “system” is the *Wolbachia*-transfected mosquito. *Wolbachia* transfection in *Wolbachia*-free mosquitoes induces oxidative stress that activates the Toll pathway through the production of ROS [122]. The subsequent production of the antimicrobial peptides (AMPs) (cecropin and defensin) modulates DENV replication [122]. *Wolbachia* can also modify host-derived miRNA expression in *w*MelPop-CLA-transfected mosquitoes resulting in DENV interference [123]. In *An. gambiae*, Carissimo et al. [124] demonstrated that the siRNA pathway is not involved in midgut antiviral defense, but instead protects the post-midgut systemic compartment, which is the site of subsequent disseminated viral infection. While *Anopheles* microbiota hampers ONNV multiplication, viral infection is positively related to the microbiota. These data indicate distinct protective mechanisms that would allow an adapted response specific to each body compartment, infection stages and pathogens.

Overall, these results highlight the need to decipher genetic and molecular mechanisms of interactions in vector pathosystems and their impacts on pathogen transmission. Complex and potentially conflicting interactions in the gut mentioned above might have some direct implications in the design of new vector control strategies based on host microbiota manipulation. Caution is necessary to avoid using mosquito colonies that could potentially host new or circulating pathogenic agents in nature.

In accordance with the recent “pathobiome” concept, i.e. the pathogenic agent integrated within its biotic environment, disease transmission is modulated by interactions between host-transmitted pathogens and commensal and mutualistic microbes [125]. The gut microbiota would not be simply a passive commensal population with limited functions but an active sensor that would contribute to a local or systemic immune response, as previously demonstrated in *Drosophila* and *Anopheles* [126–128]. The mechanisms underlying these complex multipartite interactions [host-microbial community-environment] that modulate persistence, transmission and evolution of infectious pathogens remain to be deciphered. Understanding these interactions can open new avenues for controlling transmitted pathogen infection in vector insects.

#### Mosquito-associated eukaryotes and pathogen interactions

Some yeasts can directly, or via the host, interfere with parasites. For instance, *Wickerhamomyces* produces an anti-plasmodial toxin in vitro, while *Penicillium chrysogenum* promotes *Plasmodium* infection by suppressing the host innate immune response [129, 130]. Muturi et al. [43] recently showed that the *Meyerozyma* yeast dominated fungal communities in response to LACV infections in field-collected *Ae. triseriatus* and *Ae. japonicus* females. Secreted factors by the fungus *Talaromyces* downregulate digestive enzymes of its natural host *Ae. aegypti* that modulate DENV infection [131].

#### Mosquito-borne and mosquito-specific virus interference

Evidences of interference between MSVs and MBVs are inconsistent between studies. For instance, WNV replication was reported to decrease in the *Ae. albopictus* C6/36 cell line when co-infected with a CxFV strain isolated from Colorado [62] but not with one from Guatemala [132]. The CxFV Japan strain promotes dengue and Japanese encephalitis (JEEV) virus infection in *Culex tritaeniorhynchus* cells [133]. However, replication of JEEV and WNV decreased when co-infected with the Nhumirim flavivirus (NHUV) in C6/36 cells [134, 135]. Superinfection exclusion, which corresponds to an infected cell that is refractory to be secondarily infected with another closely related (or not) virus, could explain the observed interference effects. Different combinations of mosquito cell lines, MBV and MSV strains could also explain the disparities in the reported results.

In vivo experiments are essential to corroborate the interference between MSVs and MBVs, especially since in vitro studies have recurrently used the C6/36 cell line that is deficient in the RNAi immune pathway. Nasar et al. [64] demonstrated negative effects of EILV on CHIKV replication or dissemination in both in C7/10 *Ae. albopictus* cells and *Ae. aegypti* mosquitoes, respectively (Table 1) [64].

Most other in vivo studies have focused on CFxV and WNV interactions in *Culex* sp. but with contrasting results (Table 1). West Nile virus dissemination decreased when *Cx. pipiens* were intrathoracically infected with CxFV but not with NHUV [62, 135]. The same phenotype was observed in a CxFV-positive colony from Colorado in comparison to a CxFV-negative colony from Iowa [62]. Co-inoculation of WNV and CxFV led to a reduction of WNV transmission in *Cx. quinquefasciatus* populations from Honduras while no effect was observed when the experiment was reproduced with Floridians populations [132]. Moreover, a *Cx. quinquefasciatus* NHUV-positive colony was found to transmit WNV less efficiently at days 7 and 9 post-infection (pi) (Table 1) [135]. Viral interference in *Culex sp.* thus depends on the mosquito species, MSV and MBV strain combination and/or the mosquito infection status with ISVs. Overall, genotype-by-genotype-by-genotype interactions (mosquito-MSV-MBV), the status, time and mode (natural, oral or intrathoracic) of infection, as well as intra-microbial interactions in the mosquito holobiont, could explain the differences observed in these studies.

The evolutionary and phylogenetic aspects mentioned above indicate that the function and evolution of the holobiont-associated microbiota can shape keystone phenotypes such as the vectorial capacity, a key component of the dynamic of vector-borne disease transmission. This also underscores the need to decipher factors and mechanisms involved in host restriction or permissiveness and virus interference in the development of new bio-control strategies or vaccines [65].

## Microbiota-based control strategies: promising perspectives?

To date, paratransgenesis approach using symbionts to produce molecules that inhibit pathogen development and/or transmission has been mainly restricted to *Anopheles* mosquitoes to prevent *Plasmodium* development [136, 137]. The most promising candidates are the bacteria *Pantoea agglomerans* and *Asaia* spp. [138, 139]. Paratransgenic strains of *Asaia bogorensis* were recently engineered using induced-fusion system of antiplasmodial effectors and bacterial secreted putative genes (encoding a siderophore receptor protein and YVTN beta-propeller repeat proteins) [140]. These effectors expressed in *An. stephensi* significantly inhibit *P. berghei* development [140]. Mancini et al. [139] evaluated paratransgenesis efficiency in semi-field conditions with genetically modified strains of *Asaia* and demonstrated their ability to colonize both *An. stephensi* and *An. gambiae* mosquito populations. Paratransgenesis still needs to be assessed in *Aedes* and *Culex* mosquitoes to impede arbovirus replication and/or transmission. The use and persistence in the environment of microbes that induce mosquito immune response to transmitted viruses (RNA interference) or produce toxins are currently unknown. Complementary data and semi-field studies are needed to evaluate the feasibility of these strategies to control or suppress mosquito populations. The development and implementation of paratransgenesis needs to be carefully evaluated to ensure safety issues for both humans and the environment, as reviewed in Touré et al. [141] and Gabrieli et al. [136].

Currently, phenotypes such as viral and parasite interferences induced by several *Wolbachia* strains (as *w*Mel and *w*Melpop of *Drosophila* transfected in mosquitoes) and cytoplasmic incompatibility are used to control mosquito populations [142, 143]. Field applications highlighted the efficiency of using *Wolbachia* transfected in *Ae. aegypti* to block DENV transmission [144]. Development of future *Wolbachia*-based strategies to prevent malaria transmission are promising, as the first known *Wolbachia*-*Anopheles* associations were recently identified [145, 146] and that *Plasmodium* development seems to be related to the presence of *Wolbachia* in *Anopheles coluzzii* field populations [146].

The incompatible insect technique (IIT) can also be combined with the sterile insect technique (SIT) to improve vector control [147]. For instance, Zhang et al. [148] studied the effect of *w*Pip (*Wolbachia* strain from *Cx. pipiens*) on *Ae. albopictus* that is originally naturally bi-infected by *w*AlbA and *w*AlbB. They showed that *w*Pip has a low effect on mosquito fitness. Thereby, the authors conclude that the competitiveness with natural populations combined with the strong cytoplasmic incompatibility of this triple *Wolbachia*-infected *Ae. albopictus* line supports its use in SIT/IIT strategies to control *Ae. albopictus* populations.

To conclude, field applications of these different strategies, combined or not, need properly ethical, ecological and social issues, especially if the strategies are related to the release of symbiotically modified mosquitoes [136]. Particular attention should be given to ecological and evolutionary aspects. More data will improve our understanding on the implications, the outcome and the environmental sustainability of these engineering systems, notably for potential gene flows (HGT), the emergence of resistance in mosquito populations and/or the accidental spread to non-target species. In this context, future innovative control strategies should favour the reduction of mosquito density below threshold levels of disease transmission rather than the population-replacement strategy.

## Future challenges in mosquito holobiont investigations

Some key components and processes should be determined to gain further insight into the mosquito holobiont. In particular, little is known on the (co)evolutionary aspects of mosquito holobiont functioning, including the involvement of associated-microbiota in adaptation and speciation [149, 150]. Mosquito invasive capacities and global spread could be emphasized through its microbial compartment. Mutualistic symbionts are notably a key factor for the ecological success and adaptation to novel environments of some insect species. Most striking examples are the symbiont-mediated insecticide resistance in the bean bug *Riptortus pedestris* [151] or thermal tolerance of insect aphids due to a mutation in their bacterial symbiont *Buchnera aphidicola* [152]. Besides, microbe genomes evolve relatively rapidly through mutations, recombinations, rearrangements, horizontal transfers and hybridizations. Consequently, the microbial hologenome responds differentially and more quickly to selective environmental pressure than the host’s genome. These rapidly evolving characteristics in the symbiotic community could markedly contribute to extending the host and/or their microbiota-associated phenotypes. Moreover, it is also essential to enhance knowledge on holobiont (microbiota and host) genome architecture and expression via high throughput OMICS strategies (genomics, transcriptomics, proteomics, metabolomics, etc.) in order to shape functional networks and obtain a deeper understanding on the multipartite interactions involved. Greater effort will thus be needed in developing *ad hoc* protocols and tools.

## Conclusions

The holobiont concept has raised considerable debate on the functioning and evolution of organisms with the aim of gaining greater insight into important biological issues. Host-associated microbiota involves a complex network of cooperation and competition, which makes it difficult to understand the role of each microorganism. In mosquitoes, recent findings have given rise to new hypotheses on holobiont functioning and dynamics, with symbiotic interactions being the keystone of the vector pathosystem. Intra-community microbial interaction dynamics within the mosquito holobiont as well as the molecular mechanisms involved in these multipartite interactions have yet to be investigated in detail. A technical issue is that deciphering the intricate interactions between microbes, host and environment is challenging. Such investigations could provide new targets and tools for vector-borne pathogen control. Moreover, we believe that studying the mosquito holobiont in the evolutionary context (experimentally, modeling, etc.) could help to predict, for instance, areas suitable for mosquito adaptation or even outbreaks, and thus lead to the development of strategies to avoid outbreaks, epidemics and epizootic diseases.

## Additional file

Additional file 1: List of bacteria, fungi and viruses found in different larva and adult mosquito species [15, 17, 18, 20–22, 24, 25, 27, 29, 32, 35–38, 52, 54, 61, 68, 69, 81, 103, 154–157]. List of articles mentioned are published since 2013. Previous publications on this research topic are available in our previous review [16]. (XLSX 56 kb)

## Abbreviations

AeFV: *Aedes* flavivirus
AMPs: Antimicrobial peptides
Bti: *Bacillus thuringiensis* subspecies *israelensis*
CFAV: Cell fusion agent virus
CHIKV: Chikungunya virus
CxFV: *Culex* flavivirus
DENV: Dengue virus
EILV: Eilat virus
EVEs: Endogenous viral elements
HGTs: Horizontal gene transfers
HIFs: Hypoxia-induced transcription factors
IIT: Incompatible insect technique
Imd: Immune deficiency
ISVs: Insect-specific viruses
JAK-STAT: Janus kinases and signal transducers and activators of transcription
JEEV: Japanese encephalitis
KRV: Kamiti River virus
LACV: La Crosse virus
MAMPs: Microbe-associated molecular patterns
MBVs: Mosquito-borne viruses
miRNAs: MicroRNAs
MSVs: Mosquito-specific viruses
NHUV: Nhumirim virus
ONNV: O'nyong'nyong virus
OTUs: Operational taxonomical units
PGRPs: Peptidoglycan recognition proteins
pi: Post-infection
piRNAs: PIWI interacting RNAs
PM: Peritrophic matrix
RNAi: RNA interference
SINV: Sindbis virus
SIT: Sterile insect technique
siRNA: Small interfering RNAs
TEP: Thioester-containing protein
WHO: World Health Organization
WNV: West Nile virus
YFV: Yellow fever virus
ZIKV: Zika virus
